# Identifying Hearing Loss and Audiological Rehabilitation Candidacy Through Self-Perceived Hearing Handicap Using the Croatian Version of the Hearing Handicap Inventory for the Elderly—Screening (HHIE-S-CRO)

**DOI:** 10.3390/audiolres15050116

**Published:** 2025-09-12

**Authors:** Luka Bonetti, Ana Bonetti, Tea Krišto

**Affiliations:** 1 Department of Hearing Impairments, Faculty of Education and Rehabilitation Sciences, University of Zagreb, Borongajska cesta 83f, 10000 Zagreb, Croatia; 2 Department of Speech and Language Pathology, Faculty of Education and Rehabilitation Sciences, University of Zagreb, Borongajska cesta 83f, 10000 Zagreb, Croatia; ana.bonetti@erf.unizg.hr; 3 Clinic for Rheumatology, Physical Medicine and Rehabilitation, Sestre Milosrdnice University Hospital Centre, Vinogradska cesta 29, 10000 Zagreb, Croatia; tea.kristo@kbcsm.hr

**Keywords:** age-related hearing loss, screening, self-assessment, HHIE-S questionnaire, reliability, validity

## Abstract

**Background/Objectives:** This study aimed to: (1) evaluate the effectiveness of the Croatian version of the Hearing Handicap Inventory for the Elderly—Screening Version (HHIE-S-CRO) in screening for hearing loss greater than 20 dB HL in the better-hearing ear among adults aged ≥ 60 years; (2) assess its utility in identifying individuals with hearing loss ≥ 40 dB HL in the better-hearing ear, meeting current Croatian eligibility criteria for state-funded hearing aid rehabilitation; and (3) determine whether the emotional and social components of perceived hearing handicap can be meaningfully distinguished. **Methods:** Validity of the HHIE-S-CRO was analyzed using Spearman’s correlation coefficient, the Mann–Whitney test and the factor analysis, while reliability was assessed via Cronbach’s alpha and the intraclass correlation coefficient (ICC). Receiver operating characteristic (ROC) curve analysis was calculated to determine sensitivity, specificity, and positive and negative predictive values (PPV and NPV) at various cut-off scores of the HHIE-S-CRO total for specified audiometric criteria (better ear pure-tone average > 20 dB HL and ≥40 dB HL). The nonparametric Wilcoxon Matched Pairs Test was used to compare scores on the emotional and social subscales of the HHIE-S-CRO. **Results:** The HHIE-S-CRO demonstrated excellent internal consistency (Cronbach’s alpha = 0.92) and high repeatability of the results (ICC = 0.92). Discriminant, convergent, construct and predictive validity were confirmed. The area under the curve (AUC) for detecting hearing loss > 20 dB HL in the better ear was 0.95, with a sensitivity of 90.67% and specificity of 94.65% at a cut-off score of 6. For the Croatian threshold for state-supported hearing aid rehabilitation (≥40 dB HL in the better-hearing ear), similarly favorable screening characteristics were found at a cut-off score of 10. **Conclusions:** Based on these findings, the HHIE-S-CRO appears to offer sufficient sensitivity and specificity to support two key clinical applications: (1) screening for hearing loss > 20 dB HL in individuals aged 60 and older, and (2) identifying individuals within this age group who may be eligible for state-supported hearing aid-based rehabilitation.

## 1. Introduction

According to the World Health Organization (WHO), approximately 400 million adults worldwide require rehabilitation for disabling hearing loss, defined as hearing loss greater than 35 decibels (dB) in the better-hearing ear [1]. The prevalence of hearing loss increases with age: approximately 5% of adults aged 45–54 years have disabling hearing loss, rising to 10% among those aged 55–64 years, 22% in the 65–74 age group, and up to 55% in individuals aged 75 years and older [2].

Physiological and anatomical age-related changes in the auditory system are the most common cause of hearing loss in adults, particularly in the elderly, among whom it is the third most prevalent chronic condition [3]. However, other contributing factors include genetic predispositions, hormonal influences, noise exposure, ototoxic medications, history of ear infections, and certain systemic diseases [4].

Hearing impairment hinders effective communication, thereby negatively impacting interpersonal relationships, social engagement, and economic stability. This, in turn, can lead to emotional distress and a diminished quality of life [5]. Over time, the emotional burden associated with communication difficulties due to untreated hearing loss can adversely affect psychological well-being and self-efficacy [6,7], eventually resulting in social isolation [8,9]. These cascading effects may further contribute to a decline in both mental and physical health [10,11,12,13].

Despite its high prevalence and far-reaching effects—social, emotional, psychological, mental, and physical—hearing loss often remains underdetected and consequently undertreated [14,15]. Since hearing loss in adults, and especially in the elderly, tends to be progressive [4], delayed intervention exacerbates the associated consequences [16], complicating rehabilitation and reducing its likelihood of success. Therefore, early diagnosis is essential for effective management and prevention of secondary conditions [17].

Pure-tone audiometry remains the gold standard for diagnosing hearing loss. However, screening tools are also available that can expedite the diagnostic process and indicate the need for a comprehensive audiological evaluation, which includes pure-tone audiometry.

Among these, self-report instruments—both single- and multi-item questionnaires—have gained attention as methods to assess perceived hearing ability in everyday contexts. Despite mixed findings regarding their sensitivity and specificity [18], such tools are generally considered valid, quick, and easy to administer in clinical settings and are frequently employed to identify individuals at risk.

The Hearing Handicap Inventory for the Elderly—Screening Version (HHIE-S) [19,20] is the most widely used and validated self-assessment tool for this purpose [21]. It measures the social and emotional consequences of hearing loss and demonstrates sensitivity ranging from 25% to 100% and specificity between 66% and 97%, depending on the chosen cut-off score and the audiometric definition of hearing loss [18]. The HHIE-S has been adapted into multiple languages [3,22,23,24,25,26,27,28], facilitating cross-national comparisons in hearing research.

Although Croatia has its own validated hearing screening instrument—the Hearing Self-Assessment Questionnaire [29]—adapting the HHIE-S into Croatian would enable more active participation of Croatian researchers in international studies. Furthermore, the utility of having a globally recognized screening tool for a highly prevalent and burdensome condition—with well-established, cost-effective rehabilitation options [30,31,32]—is of clear value.

Thus, the primary aim of the present study was to adapt and validate the Croatian version of the HHIE-S (HHIE-S-CRO), and to evaluate its effectiveness in detecting hearing loss greater than 20 dB HL in the better-hearing ear, in accordance with WHO guidelines [33], among individuals aged 60 years and older. Following the example of Duchêne et al. [3], the secondary aim was to assess its potential for identifying hearing loss ≥40 dB HL in the better-hearing ear, which constitutes the current threshold for state-funded hearing aid rehabilitation in Croatia [34]. Given that the HHIE-S includes items that assess both emotional and social consequences of hearing loss, the third objective was to determine whether one of these two domains has a greater influence on self-perceived hearing handicap than the other.

## 2. Materials and Methods

Data were collected at the University Hospital Center in Zagreb and the Clinical Hospital in Zagreb. Ethical approval was obtained from the respective ethics committees (Reference Numbers: 34-1/2017 and 01-1094), and the hospital administration approved a three-month data collection period.

During this period, all outpatients referred by their general practitioners for audiological evaluation were invited to participate through an informational leaflet provided by clinic staff.

Inclusion criteria were: age ≥ 60 years; referral for audiological assessment due to symptoms unrelated to hearing (e.g., throat or nasal conditions); ability to provide informed consent and complete the HHIE-S-CRO independently.

Exclusion criteria were: age < 60 years; previous audiological testing or counseling; diagnosed hearing loss; use of hearing aids; any obvious cognitive, visual, or linguistic barriers to consent or completion of the HHIE-S-CRO (e.g., difficulties in reading, comprehension, or writing). Exclusion criteria were defined in accordance with prior HHIE-S validation studies (3, 26, 28), which recruited participants without experience of hearing loss or rehabilitation to avoid bias from prior counseling, hearing aid use, or similar interventions. Excluding previously diagnosed or rehabilitated individuals minimized response bias and better reflected real-world screening conditions, where the HHIE-S-CRO is applied to both individuals with and without hearing impairment.

Eligibility was determined by clinic staff using a brief checklist and demographic information routinely collected during registration. In total, 145 participants (50 men, 95 women), aged 60–89 years (mean age: 71.65 ± 7.19 years), provided informed consent and met the study criteria.

Participants first completed the HHIE-S-CRO questionnaire independently in a quiet, well-lit room. This was followed by a standardized hearing evaluation comprising: otoscopy, performed by a specialized audiologist; tympanometry and cochleostapedial reflex testing, administered by a trained nurse; pure-tone audiometry, performed by an experienced technician in accordance with ISO 8253-1 standards [35].

The results of the audiological assessment were interpreted by a qualified audiologist.

To address the first research objective, hearing loss was defined as a better-ear pure-tone average (PTA) exceeding 20 dB HL across the frequencies 0.5, 1, 2, and 4 kHz, in line with WHO criteria [33]. Based on this definition, 75 participants (29 men and 46 women; mean age: 73.41 ± 7.28 years) were classified as having hearing loss. The mean four-frequency better-ear pure-tone average (BE PTA-4) in this group was 41.33 ± 10.61 dB HL (range: 23–69 dB HL), and the distribution of hearing loss across different age strata is presented in Table 1. The remaining 70 participants (21 men, 49 women; mean age: 69.69 ± 6.84 years) did not meet this threshold and were categorized as the hearing group.

For the second objective, hearing loss was defined as a BE PTA-4 ≥ 40 dB HL, consistent with Croatian criteria for state-funded hearing aid provision [34]. Based on this definition, 37 participants (16 men and 21 women; mean age: 74.05 ± 8.39 years) were classified as eligible for state-funded hearing aid rehabilitation. The mean BE PTA-4 in this group was 49.70 ± 7.45 dB HL (range: 41–69 dB HL). The distribution of hearing loss across different age strata for the group eligible for state-funded hearing aid rehabilitation is presented in Table 1. The remaining 108 participants (34 men, 74 women; mean age: 70.74 ± 5.65 years) did not meet this threshold and were classified as not eligible for state-funded hearing aid rehabilitation.

Table 2 presents the distribution of participants across grades of hearing loss according to the WHO [33] definition, along with basic descriptive data on sex, age, and BE PTA-4, as well as eligibility for hearing aid rehabilitation supported by state funds [34].

The HHIE-S [19,20] is a 10-item self-assessment questionnaire designed to evaluate the emotional and social/situational consequences of hearing impairment. Respondents answer each item by selecting one of three possible responses: “Yes” (4 points), “Sometimes” (2 points), or “No” (0 points). A higher total score (maximum 40 points) indicates a greater overall degree of perceived hearing handicap. The HHIE-S contains five items assessing the emotional consequences and five items assessing the situational/social consequences of hearing loss. For each of these domains, a total score can be calculated, with a maximum of 20 points.

The HHIE-S was translated into Croatian by following procedure: two independent speech-language pathologists performed forward translations, which were reconciled into a single version with input from an independent audiology specialist; the reconciled version was back-translated by a bilingual audiologist, and minor discrepancies were resolved through discussion to ensure conceptual equivalence and clarity. The final HHIE-S-CRO preserved the structure and scoring of the original questionnaire.

Descriptive analysis was conducted on the results obtained using the HHIE-S-CRO. The nonparametric Wilcoxon Matched Pairs Test was used to examine whether the emotional and social domains of the HHIE-S-CRO are differentially influenced by hearing loss. Specifically, differences in scores on the emotional and social subscales were analyzed among individuals with hearing loss greater than 20 dB HL in the better-hearing ear, as well as among participants eligible for state-funded hearing aid rehabilitation (hearing loss ≥40 dB HL in the better-hearing ear).

Reliability was evaluated using Cronbach’s alpha coefficient, with benchmarks defined as follows: >0.90 indicating excellent reliability, >0.80 good reliability, and >0.70 acceptable reliability [36]. The intraclass correlation coefficient (ICC) was calculated as an indicator of the consistency or similarity of repeated measurements, with values of 0.50–0.75 reflecting moderate reliability, 0.75–0.90 good reliability, and >0.90 excellent reliability [37].

Discriminant validity of the HHIE-S-CRO was examined using the nonparametric Mann–Whitney U test, which assessed the impact of hearing loss on self-perceived hearing handicap by testing differences in total HHIE-S-CRO scores between groups defined according to selected audiometric criteria (hearing loss in the better-hearing ear >20 dB HL or ≥40 dB HL).

Convergent validity was assessed using Spearman’s rank-order correlation to examine whether the average hearing threshold in the better-hearing ear influences the total HHIE-S-CRO score.

Construct validity was examined using factor analysis.

Predictive validity was evaluated using receiver operating characteristic (ROC) curve analysis, in which all test outcomes were classified as positive (indicative of hearing loss) or negative (non-indicative of hearing loss) according to an external criterion—the gold standard—defined in this study as audiometric thresholds of BE PTA-4 > 20 dB HL and ≥40 dB HL. The ROC curve illustrates the relationship between sensitivity (the proportion of individuals with a positive screening test score who truly have the target condition according to the reference standard) and specificity (the proportion of individuals with a negative screening test score who truly do not have the target condition). These parameters were utilized to calculate Youden’s J Index, with its maximum value identifying the cut-off score at which both sensitivity and specificity are optimized to most effectively satisfy clinical requirements.

In addition, the positive predictive value (PPV; the proportion of positive results that are true positives) and negative predictive value (NPV; the proportion of negative results that are true negatives) were calculated for both audiometric thresholds (BE PTA-4 > 20 dB HL and ≥40 dB HL). The area under the curve (AUC) was calculated as an overall measure of the HHIE-S-CRO’s screening accuracy. AUC values range from 0 (completely inaccurate test) to 1 (perfectly accurate test), with higher values indicating better accuracy. Screening accuracy was interpreted according to the following benchmarks: ≤0.50, chance-level accuracy; 0.50–0.60, very poor; 0.60–0.70, poor; 0.70–0.80, acceptable; 0.80–0.90, excellent; and ≥0.90, outstanding discriminatory ability [38].

The significance level for all statistical procedures was set at *p* < 0.05. All analyses were performed using IBM SPSS Statistics Version 29 (IBM Corporation, Armonk, NY, USA).

## 3. Results

A comparison of response frequencies for individual HHIE-S-CRO items based on the audiological criteria BE PTA-4 > 20 dB HL and BE PTA-4 ≥ 40 dB HL is presented in Table 3. It is evident that the hearing loss group defined by the BE PTA-4 ≥ 40 dB HL criterion shows a higher proportion of “Sometimes” and especially “Yes” responses compared to the group defined by the BE PTA-4 > 20 dB HL criterion. This suggests that the HHIE-S-CRO is capable of reflecting more pronounced socio-emotional difficulties, i.e., a greater hearing handicap, associated with a more stringent audiological threshold for hearing loss in the better ear.

Exceptions to this pattern include difficulties in hearing soft sounds (e.g., whispers) and arguments with family members due to hearing problems, which appear to be equally challenging under both audiological criteria.

**Figure 1:**
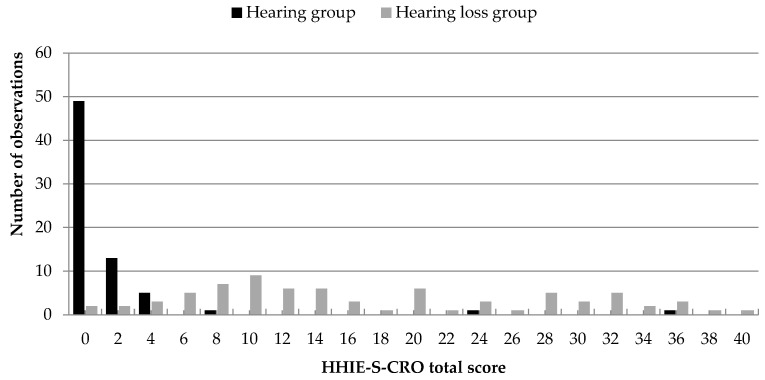
Distribution of total HHIE-S-CRO scores for normal-hearing participants and those with hearing loss, grouped according to the audiological criterion BE PTA-4 > 20 dB HL.

**Figure 2:**
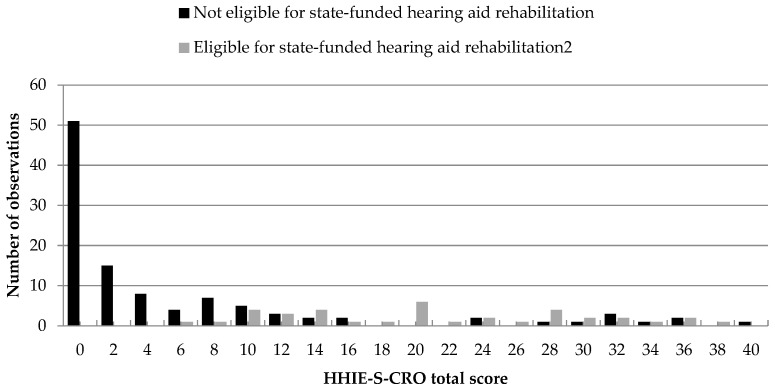
Distribution of total HHIE-S-CRO scores for participants eligible and not eligible for state-funded hearing aid rehabilitation, grouped according to the audiological criterion BE PTA-4 ≥ 40 dB HL.

Figure 1 and Figure 2 illustrate the distribution of total HHIE-S-CRO scores for participants grouped according to the audiological criteria BE PTA-4 > 20 dB HL and BE PTA-4 ≥ 40 dB HL, respectively.

Inspection of Figure 1 reveals that the results of the hearing group tend to cluster around a total HHIE-S-CRO score of 0, with smaller concentrations around scores of 2 and 4. In contrast, the hearing loss group (based on the BE PTA-4 > 20 dB HL criterion) shows a more even dispersion of total scores, suggesting a broader range of perceived hearing handicap at this lower audiological threshold.

In Figure 2, when applying the stricter criterion (BE PTA-4 ≥ 40 dB HL), the score distribution for the group eligible for state-funded hearing aid rehabilitation appears less dispersed across the scale and begins at a total score of 6, resulting in a more compressed distribution. However, approximately 30% of scores in the group not eligible for state-funded hearing aid rehabilitation fall within the range of 2 to 8 points.

Table 4 presents descriptive data on the total scores of participants with BE PTA-4 > 20 dB HL and those eligible for state-funded hearing aid rehabilitation (BE PTA-4 ≥ 40 dB HL) on the HHIE-S-CRO items assessing emotional and social consequences of hearing loss, as well as the results of the Wilcoxon Matched Pairs Test. In both participant groups, the total score on the social subscale was significantly higher than that on the emotional subscale (*p* ≤ 0.001 and *p* = 0.004, respectively), suggesting that the social consequences of hearing loss are perceived as more impactful in everyday situations.

**Figure 3:**
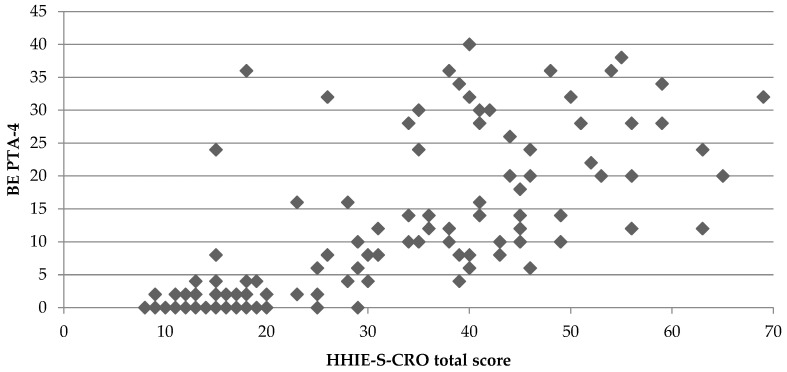
Scatterplot of the total HHIE-S-CRO scores in relation to the four-frequency better ear pure-tone average (BE PTA-4).

Figure 3 displays the scatterplot of total HHIE-S-CRO scores in relation to the four-frequency better ear pure-tone average (BE PTA-4). The plot reveals a clear trend of increasing total HHIE-S-CRO scores with higher BE PTA-4 values, indicating a positive correlation between the two variables. This visual suggestion was confirmed by a Spearman correlation analysis, which showed a strong, positive, and statistically significant association between total HHIE-S-CRO scores and BE PTA-4 (ρ = 0.80, *p* < 0.01). These results support the convergent validity of the HHIE-S-CRO.

**Figure 4:**
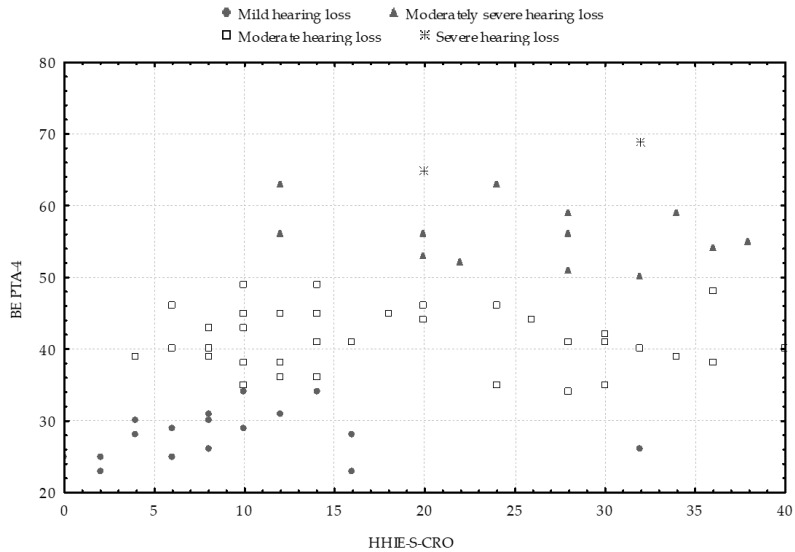
Scatterplot of total HHIE-S-CRO scores in relation to the four-frequency better-ear pure-tone average (BE PTA-4), presented according to hearing loss grades [33].

Figure 4 displays the scatterplot of total HHIE-S-CRO scores in relation to the hearing loss grades [33]. The plot reveals clustering of scores among participants whose BE PTA-4 falls within the category of mild hearing loss, predominantly within the 0–15 point range on the HHIE-S-CRO. In contrast, the scores of participants with higher grades of hearing loss extend across nearly the entire scale: participants with moderate hearing loss scored between 4 and 40 points, while those with moderately severe hearing loss scored between 12 and 38 points. Overlaps in total HHIE-S-CRO scores are also clearly visible across all hearing loss grades.

The reliability of the HHIE-S-CRO was assessed using Cronbach’s alpha coefficient as a measure of internal consistency. For the overall HHIE-S-CRO, the coefficient was 0.92, while the values for the social and emotional subscales were 0.83 and 0.86, respectively, indicating excellent reliability for the full scale and good reliability for the subscales [36]. Item–total correlations ranged from 0.53 to 0.77, and Cronbach’s alpha if item deleted varied between 0.90 and 0.92. The ICC was 0.92 (95% CI: 0.89–0.94), further supporting the excellent reliability of the HHIE-S-CRO [37].

The results of the nonparametric Mann–Whitney U test indicated a statistically significant difference in total HHIE-S-CRO scores between hearing and hearing loss groups, based on the selected audiometric criteria. These results are presented in Table 5 and demonstrate that the HHIE-S-CRO possesses discriminant validity.

Factor analysis was supported by a high The Kaiser–Meyer–Olkin value (0.914) and significant Bartlett’s test of sphericity (χ^2^ = 824.96, df = 45, *p* < 0.001). Principal axis factoring yielded a single factor (only one eigenvalue >1, i.e., 5.78) explaining 57.83% of the total variance. The factor loadings presented in Table 6 indicate that all HHIE-S-CRO items have a strong association with the factor, supporting its construct validity.

**Figure 5:**
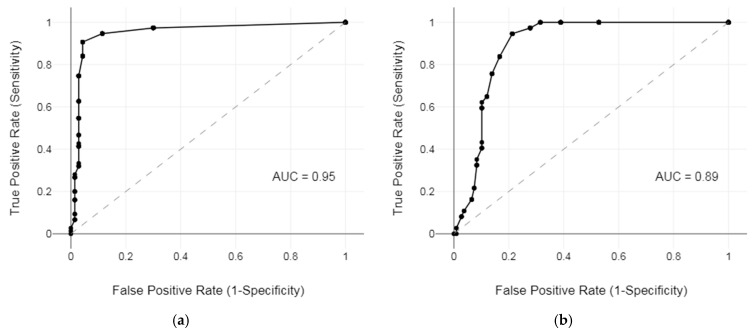
ROC curve analysis was conducted to evaluate the predictive validity of the HHIE-S-CRO under two audiometric criteria: BE PTA-4 > 20 dB HL (**a**) and BE PTA-4 ≥ 40 dB HL (**b**).

ROC curve analysis was conducted to evaluate the predictive validity of the HHIE-S-CRO under two audiometric criteria: BE PTA-4 > 20 dB HL and BE PTA-4 ≥ 40 dB HL (Figure 5). For the first criterion (BE PTA-4 > 20 dB HL), AUC was 0.95 (95% CI: 0.91–0.99), indicating outstanding discrimination between individuals with and without hearing loss at a cut-off score of 6 (a total HHIE-S-CRO score <6 indicated the absence of hearing loss, and a total HHIE-S-CRO score ≥6 indicated the presence of hearing loss). At this threshold, the scale demonstrated excellent predictive performance, with sensitivity of 90.67% (95% CI: 81.71–96.16%), specificity of 95.71% (95% CI: 87.98–99.11%), positive predictive value (PPV) of 95.77% (95% CI: 88.20–98.57%), and negative predictive value (NPV) of 90.54% (95% CI: 82.52–95.10%).

For the second criterion (BE PTA-4 ≥ 40 dB HL), the AUC was 0.89 (95% CI: 0.84–0.94), with the optimal cut-off score identified as 10 (a total HHIE-S-CRO score ≤10 indicated not eligible for state-funded hearing aid rehabilitation, and a total HHIE-S-CRO score >10 indicated eligible for state-funded hearing aid rehabilitation), and at this threshold, the scale again demonstrated excellent predictive validity, with sensitivity of 83.78% (95% CI: 67.99–93.81%), specificity of 83.33% (95% CI: 74.94–89.81%), PPV of 63.27% (95% CI: 52.47–72.88%), and NPV of 93.75% (95% CI: 87.77–96.91%).

## 4. Discussion

All statistical analyses conducted support the conclusion that the HHIE-S-CRO is a successfully adapted instrument suitable for clinical use.

The ability of the HHIE-S-CRO to accurately identify individuals aged 60 and above with hearing loss greater than 20 dB HL in the better-hearing ear appears to be well-supported by the statistical findings. The scale demonstrated high reliability, with excellent internal consistency (Cronbach’s alpha of 0.92) and high repeatability of the results (ICC of 0.92). These metrics underscore the coherence of the HHIE-S-CRO and confirm the consistent contribution of individual items to the total score, as well as the total score’s relevance in assessing the risk of hearing loss greater than 20 dB HL.

Further supporting evidence includes the scale’s confirmed discriminative, convergent, construct and predictive validity, affirming its capacity to reliably quantify the socio-emotional difficulties associated with hearing loss. The scale’s predictive accuracy at the BE PTA-4 > 20 dB HL criterion was particularly high, with an AUC of 0.951, corresponding to an overall prediction accuracy of 89.66% (95% CI: 83.51–94.09%). These promising results advocate for the broader application of the HHIE-S-CRO, especially given the clinical relevance of this audiometric criterion due to the progressive nature of hearing loss in older adults [4], and the imperative for early diagnosis and intervention to maximize patient outcomes [16,17].

In this context, the high sensitivity (90.67%) and specificity (94.65%) of the HHIE-S-CRO in detecting mild hearing impairment are highly favorable. Interestingly, these excellent diagnostic properties were observed at a slightly lower cut-off score (score 6) than the more commonly cited cut-off of 8 in the literature, although earlier studies have employed cut-offs ranging from 6 to 18, yielding varying sensitivity and specificity values [39] (p. 8). Based on the presented data, it can be concluded that the primary aim of this study—namely, the adaptation and validation of the HHIE-S-CRO, as well as the evaluation of its effectiveness in detecting hearing loss greater than 20 dB HL in the better-hearing ear, in accordance with WHO guidelines [33], among individuals aged 60 years and older—has been achieved.

Similarly favorable results were observed for the second audiometric criterion (BE PTA-4 ≥ 40 dB HL), which in Croatia is the threshold for state-supported auditory rehabilitation with hearing aids. The score distribution in Figure 2 demonstrates that the HHIE-S-CRO is sensitive to detecting self-perceived hearing handicap even among individuals with mild hearing loss (BE PTA-4 > 20–40 dB HL) and accurately captures the upward shift in the minimum perceived handicap as the degree of audiometrically defined hearing loss increases (i.e., under the BE PTA-4 ≥ 40 dB HL criterion). The proportion of accurate predictions in identifying individuals eligible for audiological rehabilitation was 83.45% (95% CI: 76.38–89.10%), with an excellent AUC of 0.89 and high sensitivity (83.78%) and specificity (83.33%) at a cut-off score of 10. Based on this data, it can be considered that the second aim of this study—namely, to assess the potential of the HHIE-S-CRO for identifying hearing loss ≥40 dB HL in the better-hearing ear, which represents the current threshold for state-funded hearing aid rehabilitation in Croatia [34]—has also been achieved.

In summary, the data from this study indicate that the HHIE-S-CRO possesses sufficient sensitivity and specificity to support two essential areas of clinical decision-making: (1) screening for hearing loss exceeding 20 dB HL in adults aged ≥60 years, and (2) identifying individuals who meet the national eligibility criteria for state-funded hearing aid rehabilitation.

During the administration of the HHIE-S-CRO, no objections or difficulties were reported by the hospital staff involved in data collection, nor were any issues reported by respondents, indicating that the questionnaire items are both comprehensible and culturally appropriate for the Croatian population. Considering all previously presented statistical indicators of its clinical potential, and the fact that the administration of the HHIE-S-CRO was completed without any reported challenges or complications, it is reasonable to conclude that, in its current form, the HHIE-S-CRO is ready for evaluation of its broader integration into routine clinical practice. This is particularly relevant for assessing its performance in primary healthcare settings, such as general practitioners’ offices, where early decisions regarding referrals for audiological evaluation are made.

Despite the highly favorable screening characteristics of the HHIE-S-CRO, caution is warranted when interpreting its results. Self-assessment of hearing handicap does not constitute a diagnostic procedure, nor does it perfectly correlate with audiometrically established hearing thresholds [3] (p. 201).

As illustrated in Figure 4, the scores of participants with mild hearing loss exhibit more pronounced clustering compared to those with moderate and moderately severe hearing loss. This suggests a relatively uniform perception of hearing handicap at lower levels of hearing loss in the better ear, and a greater variability in the socio-emotional consequences as the degree of hearing loss increases. Overlaps in total HHIE-S-CRO scores are observed across all hearing loss grades, with score ranges in the moderate and moderately severe categories spanning more than 30 and 20 points, respectively.

Hence, the degree of hearing loss represents only one of several contributing factors shaping an individual’s perception of hearing difficulties. This perception is further modulated by non-audiological variables such as lifestyle, age, marital/family status, general health, education, cultural background, and mental status [40,41,42,43]. Therefore, individuals who obtain a total HHIE-S-CRO score of ≥6 should be advised to monitor for potential progression of hearing difficulties and referred for periodic audiometric evaluations. Decision-making regarding the content and thematic components of counseling can be supported by data collected in pursuit of the third objective of this study: to determine whether the emotional or social consequences of hearing loss have a greater impact on the perception of hearing handicap. Since the results of the Wilcoxon Matched Pairs Test indicated that participants, at both audiometric criteria (BE PTA-4 > 20 dB HL and BE PTA-4 ≥ 40 dB HL), demonstrated significantly higher total scores on the social subscale compared to the emotional subscale, counseling should therefore primarily address the social consequences of hearing loss, as these are perceived as more impactful in everyday situations. Given that the social consequences of hearing difficulties were perceived as significantly more impactful than their emotional counterparts, which is consistent with observations reported in other studies [28,44,45], counseling should primarily focus on recognizing early signs of declining hearing function in characteristically challenging social situations—such as conversations in noisy environments like gatherings and group discussions, or telephone communication [46,47,48]. The significantly higher total score on the social subscale of the HHIE-S-CRO suggests that, in counseling contexts, an effective strategy may be to prioritize the identification of difficulties encountered in real-life listening situations as key challenges and complaints associated with hearing loss. Finally, those scoring > 6 should be referred for formal audiometric assessment, with those scoring > 10 likely requiring audiological rehabilitation, including the provision of hearing aids.

One limitation affecting the screening utility of the HHIE-S-CRO in this study is the recruitment of participants who had already been referred for audiometric evaluation. This introduces a potential selection bias and may affect the observed prevalence of hearing loss within the study sample. Nevertheless, the context in which the HHIE-S-CRO was administered closely resembles conditions considered optimal for hearing loss screening in older adults—specifically, the use of a brief self-assessment questionnaire in a clinical setting [3]. Therefore, it is reasonable to conclude that the HHIE-S-CRO could be effectively utilized in screening a larger segment of the elderly population.

A second limitation of this study is the absence of formal content validity assessment, such as expert Content Validity Index, and the lack of cognitive pre-testing. Access to these data would have permitted verification of the HHIE-S-CRO’s representativeness through expert evaluation and ensured that respondents correctly interpreted the translated items.

A further limitation pertains to gender imbalance: the sample comprised 95 women and only 50 men, yielding a female-to-male ratio of 0.53. In contrast, the population ratio for individuals over 60 years of age in Croatia is approximately 0.74 in favor of women [49]. Consequently, further validation of the HHIE-S-CRO on a more representative sample is advisable before recommending its widespread use in large-scale screening programs.

Finally, participants in this study were volunteers who were fully independent and in relatively good general health, which limits the generalizability of the findings to other subgroups of older adults. These may include individuals who are unable to provide data due to hospitalization, institutionalization, limited or no access to healthcare services, or those who may be less motivated to manage their hearing health due to comorbidities with greater impact on quality of life, or generally modest communication needs [39].

## 5. Conclusions

The Croatian adaptation of the HHIE-S questionnaire (HHIE-S-CRO) is a reliable, valid, and cost-effective instrument that can be used to screen for hearing loss greater than 20 dB HL in the better ear among individuals aged 60 years and older. Additionally, for individuals without an indication for further audiometric hearing assessment, the tool may serve to monitor the potential progression of hearing difficulties by tracking their social consequences, as well as to identify candidates for state-funded hearing aid provision, defined as individuals with hearing loss ≥40 dB HL in the better ear.

## Figures and Tables

**Table 1:** Distribution of hearing loss across different age groups.

Age Group	Means and Standard Deviations of Audiometric Measures		
	Hearing Participants (BE PTA-4 * ≤ 20 dB HL; N = 70)	Participants with Hearing Loss (BE PTA-4 * > 20 dB HL; N = 75)	Participants with Hearing Loss Eligible for State-Funded Hearing Aid Rehabilitation (BE PTA-4 * ≥ 40 dB HL; N = 37)
60–69 years	M = 14.89 dB HL (±3.21) Range: 8–20 dB HL N = 38 (26 females and 12 males)	M = 42.21 dB HL (±9.42) Range: 23–69 dB HL N = 19 (10 females and 9 males)	M = 47.45 dB HL (±7.92) Range: 41–69 dB HL N = 11 (5 females and 6 males)
70–79 years	M = 14.92 dB HL (±3.05) Range: 9–20 dB HL N = 24 (18 females and 6 males)	M = 38.83 dB HL (±9.90) Range: 25–63 dB HL N = 42 (28 females and 14 males)	M = 49.13 dB HL (±6.70) Range: 41–63 dB HL N = 16 (11 females and 5 males)
80–89 years	M = 16.36 dB HL (±2.12) Range: 9–20 dB HL N = 8 (5 females and 3 males)	M = 47.64 dB HL (±10.98) Range: 23–65 dB HL N = 14 (8 females and 6 males)	M = 53.10 dB HL (±6.80) Range: 45–65 dB HL N = 10 (5 females and 5 males)

* Four-frequency better ear pure-tone average.

**Table 2:** Distribution of participants across grades of hearing loss according to the WHO [33] definition, along with basic descriptive data on sex, age, and BE PTA-4, as well as eligibility for hearing aid rehabilitation supported by state funds [34].

Mild hearing loss (BE PTA-4 20 to <35 dB HL)	All participants within this hearing loss grade were not eligible for hearing aid rehabilitation supported by state funds under Croatian national criteria BE PTA-4 * ≥ 40 dB HL: N = 20 (12 females and 8 males) Mean BE PTA-4: 28.70 (±3.52; range: 23–34 dB HL) Mean age: 73.45 (±4.24; range 65–84 years)
Moderate hearing loss (BE PTA-4 35 to <50 dB HL)	All participants within this hearing loss grade: N = 40 (26 females and 14 males) Mean BE PTA-4: 41.48 (±3.89; range: 35–49 dB HL) Mean age: 72.25 (±6.89; range 60–83 years) Participants within this hearing loss grade not eligible for hearing aid rehabilitation supported by state funds under Croatian national criteria BE PTA-4 * ≥ 40 dB HL: - N = 18 (13 females and 5 males) - Mean BE PTA-4: 37.83 (±1.57; range: 35–39 dB HL) - Mean age: 71.56 (±7.07; range 60–82 years) Participants within this hearing loss grade eligible for hearing aid rehabilitation supported by state funds under Croatian national criteria BE PTA-4 * ≥ 40 dB HL: - N = 22 (19 females and 9 males) - Mean BE PTA-4: 44.45 (±2.39; range: 41–49 dB HL) - Mean age: 72.59 (±7.19; range 60–88 years)
Moderately severe hearing loss (BE PTA-4 50 to <65 dB HL)	All participants within this hearing loss grade were eligible for hearing aid rehabilitation supported by state funds under Croatian national criteria BE PTA-4 * ≥ 40 dB HL: N = 13 (6 females and 7 males) Mean BE PTA-4: 55.92 (±3.99; range: 50–63 dB HL) Mean age: 78.08 (±7.59; range 65–89 years)
Severe hearing loss (BE PTA-4 65 to <80 dB HL)	All participants within this hearing loss grade were eligible for hearing aid rehabilitation supported by state funds under Croatian national criteria BE PTA-4 * ≥ 40 dB HL: N = 2 (2 females) Mean BE PTA-4: 67.00 (±2.00; range 65–69 dB HL) Mean age: 71.00 (±11.00; range 60–82 years)

* Four-frequency better ear pure-tone average.

**Table 3:** Frequency of responses to individual HHIE-S-CRO items for hearing participants/participants not eligible for state-funded hearing aid rehabilitation and participants with hearing loss/eligible for state-funded hearing aid rehabilitation, according to selected audiometric criteria and those with hearing loss, grouped according to the audiological criteria BE PTA-4 > 20 dB HL and BE PTA-4 ≥ 40 dB HL.

		BE PTA-4 * > 20 dB HL Criteria		BE PTA-4 * ≥ 40 dB HL Criteria	
**HHIE-S-CRO Item (English/Croatian)** **E—Emotional Item** **S—Social Item**	Answer	Hearing Group (N = 70)	Hearing Loss Group (N = 75)	Not Eligible for State-Funded Hearing Aid Rehabilitation (N = 108)	Eligible for State-Funded Hearing Aid Rehabilitation (N = 37)
1. (E) Does a hearing problem cause you to feel embarrassed when meeting new people? (Osjećate li se neugodno zbog slušnih problema kada upoznajete nove ljude?)	No	98.57%	38.67%	85.05%	18.42%
	Sometimes	-	24.00%	5.61%	31.58%
	Yes	1.43%	37.33%	9.35%	50.00%
2. (E) Does a hearing problem cause you to feel frustrated when talking to members of your family? (Čine li Vas slušni problemi frustriranima kada razgovarate s članovima obitelji?)	No	95.71%	40.00%	79.44%	31.58%
	Sometimes	1.43%	30.67%	10.28%	34.21%
	Yes	2.86%	29.33%	10.28%	34.21%
3. (S) Do you have difficulty hearing when someone speaks in a whisper? (Imate li poteškoća sa slušanjem kada netko šapće?)	No	72.86%	10.67%	52.34%	7.89%
	Sometimes	22.86%	8.00%	16.82%	10.53%
	Yes	4.28%	81.33%	30.84%	81.58%
4. (E) Do you feel handicapped by a hearing problem? (Osjećate li se zbog slušnih problema hendikepiranima?)	No	97.14%	64.00%	88.78%	55.26%
	Sometimes	-	20.22%	4.68%	26.32%
	Yes	2.86%	16.00%	6.54%	18.42%
5. (S) Does a hearing problem cause you difficulty when visiting friends, relatives, or neighbors? (Uzrokuju li Vam slušni problemi teškoće kada posjećujete prijatelje, rodbinu ili susjede?)	No	97.14%	58.67%	89.72%	42.11%
	Sometimes	-	9.33%	1.87%	13.15%
	Yes	2.86%	32.00%	8.41%	44.74%
6. (S) Does a hearing problem cause you to attend religious services less often than you would like? (Prisustvujete li zbog slušnih problema bogoslužju rjeđe nego što biste voljeli?)	No	100%	78.67%	94.39%	73.68%
	Sometimes	-	2.66%	-	5.26%
	Yes	-	18.67%	5.61%	21.06%
7. (E) Does a hearing problem cause you to have arguments with family members? (Uzrokuju li Vam slušni problemi prepirke s članovima obitelji?)	No	97.14%	57.33%	84.11%	55.26%
	Sometimes	-	17.33%	5.61%	18.42%
	Yes	2.86%	25.34%	10.28%	26.32%
8. (S) Does a hearing problem cause you difficulty when listening to TV or radio? (Uzrokuju li Vam slušni problemi teškoće pri slušanju televizije ili radija?)	No	90.00%	29.33%	75.70%	10.52%
	Sometimes	8.57%	22.67%	11.21%	28.95%
	Yes	1.43%	48.00%	13.09%	60.53%
9. (E) Do you feel that any difficulty with your hearing limits or hampers your personal or social life? (Smatrate li da neka poteškoća s Vašim slušanjem ograničava ili sputava Vaš osobni ili društveni život?)	No	94.28%	57.33%	85.98%	44.74%
	Sometimes	2.86%	17.33%	5.61%	23.68%
	Yes	2.86%	25.34%	8.41%	31.58%
10. (S) Does a hearing problem cause you difficulty when in a restaurant with relatives or friends? (Uzrokuju li Vam slušni problemi teškoće kada ste u restoranu s rodbinom ili prijateljima?)	No	97.14%	45.33%	83.18%	34.21%
	Sometimes	-	22.67%	4.67%	31.58%
	Yes	2.86%	32.00%	12.15%	34.21%

* Four-frequency better ear pure-tone average.

**Table 4:** Results of the nonparametric Wilcoxon Matched Pairs Test used to examine differences in total scores between the emotional and social subscales of the HHIE-S-CRO, based on selected audiometric criteria.

Audiometric Criteria	HHIE-S-CRO Subscale		Wilcoxon Matched Pairs Test		
			T	Z	*p*
BE PTA-4 > 20 dB HL (N = 75)	Emotional	Mean = 7.39 (±5.95) Range: 0–20	392.50	4.214	<0.001
	Social	Mean = 9.76 (±5.55) Range: 0–20			
BE PTA-4 ≥ 40 dB HL (N = 37)	Emotional	Mean = 9.30 (±4.97) Range: 0–18	120.50	2.859	0.004
	Social	Mean = 11.57 (±5.15) Range: 2–20			

**Table 5:** The results of the nonparametric Mann–Whitney U test used to test differences in HHIE-S-CRO total scores between participants with and without hearing impairment, and participants eligible/not eligible for state-funded hearing aid rehabilitation according to selected audiometric criteria.

Audiometric Criteria		HHIE-S-CRO		
		Rank Sum	U	*p*
BE PTA-4 > 20 dB HL	Hearing group (N = 70)	2740.00	255	<0.001
	Hearing loss group (N = 75)	7845.00		
BE PTA-4 ≥ 40 dB HL	Eligible for state-funded hearing aid rehabilitation (N = 108)	4255.50	443.50	<0.001
	Not eligible for state-funded hearing aid rehabilitation (N = 37)	6329.50		

**Table 6:** Assessment of construct validity of HHIE-S-CRO: results of factor analysis and loadings of the single-factor solution extracted using the principal axis factoring method.

	Factor
1. (E) Does a hearing problem cause you to feel embarrassed when meeting new people?	0.81
2. (E) Does a hearing problem cause you to feel frustrated when talking to members of your family?	0.71
3. (S) Do you have difficulty hearing when someone speaks in a whisper?	0.64
4. (E) Do you feel handicapped by a hearing problem?	0.74
5. (S) Does a hearing problem cause you difficulty when visiting friends, relatives, or neighbors?	0.83
6. (S) Does a hearing problem cause you to attend religious services less often than you would like?	0.55
7. (E) Does a hearing problem cause you to have arguments with family members?	0.70
8. (S) Does a hearing problem cause you difficulty when listening to TV or radio?	0.71
9. (E) Do you feel that any difficulty with your hearing limits or hampers your personal or social life?	0.77
10. (S) Does a hearing problem cause you difficulty when in a restaurant with relatives or friends?	0.80

## Data Availability

The data presented in this study are available on request from the corresponding author due to privacy, legal and ethical reasons.

## Abbreviations

The following abbreviations are used in this manuscript:

HHIE-S-CRO	Croatian adaptation of HHIE-S
dB HL	Decibels hearing level
ROC curve	Receiver operating characteristic curve
PPV	positive predictive values
NPV	negative predictive values
ICC	Intraclass correlation coefficient
AUC	Area under the curve
WHO	World Health Organization
HHIE-S	The Hearing Handicap Inventory for the Elderly—Screening Version
PTA	Pure tone average
BE PTA-4	Four frequency better ear PTA
